# Neurophysiological signatures of sensory-processing sensitivity

**DOI:** 10.3389/fnins.2023.1200962

**Published:** 2023-07-20

**Authors:** Nicole Meinersen-Schmidt, Nike Walter, Patricia Kulla, Thomas Loew, Thilo Hinterberger, Joachim Kruse

**Affiliations:** ^1^Department for Clinical Psychology and Trauma Therapy, University of the Bundeswehr Munich, Neubiberg, Germany; ^2^Section of Applied Consciousness Sciences, Department of Psychosomatic Medicine, University Hospital of Regensburg, Regensburg, Germany

**Keywords:** sensory-processing sensitivity, EEG, global activity, power spectral density, diagnostics

## Abstract

**Background:**

Sensory processing sensitivity is mainly captured based on questionnaires and it’s neurophysiological basis is largely unknown. As hitherto no electroencephalography (EEG) study has been carried out, the aim of this work was to determine whether the self-reported level of SPS correlates with the EEG activity in different frequency bands.

**Methods:**

One hundred fifteen participants were measured with 64-channel EEG during a task-free resting state. After artifact correction, a power spectrum time series was calculated using the Fast Fourier Transform (FFT) for the following frequency bands: Delta: 1–3.5 Hz, theta: 4–7.5 Hz, alpha1: 8–10 Hz, alpha2: 10.5–12 Hz, beta1: 12.5–15 Hz, beta2: 15.5–25 Hz, gamma: 25.5–45 Hz, global: 1–45 Hz. Correlations with the ‘Highly Sensitive Person Scale’ (HSPS-G) scores were determined. Then, the lowest and the highest 30% of the cohort were contrasted as polar opposites. EEG features were compared between the two groups applying a paired two-tailed *t*-test.

**Results:**

The HSPS-G scores correlated statistically significantly positive with beta 1 and 2, and global EEG power during resting with eyes open, but not during resting with eyes closed. The highly sensitive group revealed higher beta power (4.38 ± 0.32 vs. 4.21 ± 0.17, *p* = 0.014), higher gamma power (4.21 ± 0.37 vs. 4.00 ± 0.25, *p* = 0.010), and increased global EEG power (4.38 ± 0.29 vs. 4.25 ± 0.17, *p* = 0.041). The higher EEG activity in the HSP group was most pronounced in the central, parietal, and temporal region, whereas lower EEG activity was most present in occipital areas.

**Conclusion:**

For the first time, neurophysiological signatures associated with SPS during a task free resting state were demonstrated. Evidence is provided that neural processes differ between HSP and non-HSP. During resting with eyes open HSP exhibit higher EEG activity suggesting increased information processing. The findings could be of importance for the development of biomarkers for clinical diagnostics and intervention efficacy evaluation.

## Introduction

Over the past two decades, research has extensively explored theoretical frameworks related to individual differences in processing sensory stimuli. One construct that has emerged during this time is sensory-processing sensitivity (SPS), which encompasses perceptual sensitivity, cognitive responses, and emotional reactions to environmental stimuli (Pluess, 2015; Turjeman-Levi and Kluger, 2022). The development of the Highly Sensitive Person Scale (HSPS) by Aron and Aron in 1997 marked an initial step in measuring high sensitivity through a 27-item questionnaire (Aron and Aron, 1997). Subsequent qualitative and quantitative studies have examined this scale (Smolewska et al., 2006; Pluess, 2015; Lionetti et al., 2019). Factor analysis conducted by Smolewska et al. revealed three factors: Ease of Excitation (EOE), Low Sensory Threshold (LST), and Aesthetic Sensitivity (AES), reflecting individuals’ susceptibility to being overwhelmed by stimuli, experiencing unpleasant sensory arousal, and being deeply moved by arts or music, respectively (Smolewska et al., 2006; Pluess, 2015; Lionetti et al., 2019).

EOE and LST have been linked to negative emotionality, anxiety, and depression, while AES has been associated with positive emotionality, openness to experience, conscientiousness, positive affect, and self-esteem (Liss et al., 2008; Ahadi and Basharpoor, 2010; Sobocko and Zelenski, 2015). Initially, Aron and Aron conceptualized SPS as a categorical trait, identifying individuals scoring high on SPS as Highly Sensitive Persons (HSPs; Aron and Aron, 1997). It is estimated that approximately 20–30% of the general population possess heightened sensory sensitivity (Aron et al., 2012; Lionetti et al., 2018; Pluess et al., 2018). A latent class analysis conducted by Lionetti et al. on HSPS results from two samples (*n* = 451 and *n* = 540) identified a low, medium, and highly sensitive group, with distributions of 29, 40, and 31% (Lionetti et al., 2018). Alternatively, researchers have proposed SPS as a temperament trait characterized by increased depth of information processing, heightened awareness of environmental subtleties, and susceptibility to overstimulation (Aron et al., 2012; Homberg et al., 2016; Greven et al., 2019). This conceptualization draws from Gray’s (1981) behavioral inhibition system (BIS), which involves pausing to evaluate behavior in response to environmental conditions (Gray, 1981). Consequently, HSPs are more inclined to carefully analyze novel situations before making decisions and taking action (Smolewska et al., 2006; Sobocko and Zelenski, 2015). The more sensitive an individual’s BIS, the more sensitive they are to new stimuli (Aron and Aron, 1997). Higher levels of SPS have been associated with mental illnesses such as anxiety, depression, and somatoform disorders (Liss et al., 2005, 2008; Bakker and Moulding, 2012; Jonsson et al., 2014; Greven et al., 2019). A twin study examining the heritability of SPS found that 47% of the variance could be explained by genetic factors (Assary et al., 2021). Additionally, Aron et al. (2005) discovered that HSPs exhibit negative affectivity and shyness under adverse environmental conditions, which are risk factors for the development of mental illnesses (Aron et al., 2005). Furthermore, research indicates that HSPs often report more stressful experiences due to their heightened perception of stimuli and deeper processing. It has been suggested that the thalamic filter, responsible for filtering out irrelevant information, identifies more stimuli as relevant in HSPs, potentially leading to increased stress (Benham, 2006; Evans and Rothbart, 2008; Jagiellowicz et al., 2011; Gerstenberg, 2012).

Although questionnaires and behavioral observational assessments have primarily captured SPS, only a limited number of fMRI studies (Jagiellowicz et al., 2011; Acevedo et al., 2014, 2018, 2021; Schaefer et al., 2022) have explored its neurobiological basis. To date, no electroencephalography (EEG) study has investigated the neurophysiological correlates of SPS, and thus, there is a lack of empirical neurophysiological markers to identify the level of SPS. Such markers could have significant implications for clinical diagnostics, enabling differentiation between psychopathologies and monitoring the effectiveness of therapeutic interventions. Additionally, the notion of enhanced information processing in SPS remains theoretical and has not been experimentally confirmed. It is hypothesized that this trait is reflected in increased electrophysiological activity, particularly in gamma oscillations, which are associated with cognitive processing and the temporal binding of perceptual stimuli. To address this hypothesis and shed light on the neurophysiological mechanisms of SPS, the present study aimed to (1) examine the correlation between self-reported SPS levels and EEG activity, and (2) identify differences in power spectral density between participants with low and high levels of SPS.

## Methods

### Participants

The participants were recruited throughout Germany via various social networks, forums, the Research Association for sensory-processing sensitivity, and internal university invitation notifications. An amount of 30 euros was offered as an incentive to participate in the study. Psychology students received subject hours. Participation was accepted from the age of 18 years. Exclusion criteria were known epilepsy, acute self-harm, acute suicidality and substance dependence. All 115 participants signed an informed consent before participating in the laboratory study.

### Behavioral assessment

Before EEG the recording all participants filled in the questionnaire ‘Highly Sensitive Person Scale’ (HSPS-G; Konrad and Herzberg, 2019). The HSPS-G (HSP scale, original version; Aron and Aron, 1997); German version Konrad and Herzberg (2019) is a 26-item self-reported questionnaire that measures the degree of sensitivity in a 5-point Likert rating scale (“0” does not apply at all – “4” applies completely). For this purpose, the measurement instrument is divided into the subscales of *Ease of Excitation* (EOE), *Aesthetic Sensitivity* (AES), and a *Low Sensory Threshold* (LST). The HSPS-G was normed and standardized on individuals from the general population and was found to have good reliability (Cronbach’s α of 0.93 to 0.95; Konrad and Herzberg, 2019).

### EEG recording

The laboratory surveys took place in a sound- and magnetic field-isolated cabin from 03.05. to 02.07.2021 on the campus of the University of the Bundeswehr in Munich. Measurement times were between 8.00 a.m. and 3 p.m. Electrophysiological data was recorded using a 72 channels QuickAmp amplifier system (BrainProducts GmbH, Munich, Germany). EEG was measured with a 64-channel ANT Waveguard electrode cap (ANT B.V., Enschede, The Netherlands) with active shielding and Ag/AgCl electrodes, which were arranged according to the international 10/10 system. Vertical electrooculograms (EOGs) were recorded above and below one eye. Electrode impedances were kept below 10 kΩ. Data was sampled at 250 Samples/s in a range from DC to 70 Hz with a notch filter at 50 Hz. Data was acquired during a task-free resting state with eyes closed and eyes open for a duration of 3 min each.

### EEG preprocessing

Matlab Version R2023a (MathWorks, Natrick, USA), Brain Vision Analyser 2.0 (Brain Products, Gilching, Germany) and SPSS Version 28 (IBM SPSS Statistical Package 28.0, IBM Corporation, Armonk, NY, USA) were used for data analysis.

After detrending the 64 EEG channels a correction for eye movement was done using a linear correction algorithm. This algorithm identifies occurrences of eye blinks and movement events, utilizing these periods to calculate a correction factor for each channel. The electrooculogram (EOG) is then multiplied by this factor and subsequently subtracted from the electroencephalogram (EEG). It has been evaluated and proven to effectively operate in normal EEG signals without movement artifacts, demonstrating satisfactory performance (Hinterberger et al., 2003). Additionally, the Independent Component Analysis (ICA) as implemented in the Brain Vision Analyser was applied using the standard protocol to identify and reject data containing muscular activity. Additionally, EEG artifact rejection was carried out with the EEGLAB plugin *clean_rawdata*. Bad channels were removed if (i) it was flat for more than 5 s, (ii) had a low signal to noise ratio with standard deviation above 4, or (iii) was poorly correlated with nearby channels (threshold: 0.8). The time-series of the raw data was then visually inspected for continuous artifacts.

### EEG power spectral density analysis

Power spectral density was calculated using the Fast Fourier Transform (FFT) algorithm (Nussbaumer, 1981). FFT was calculated on 2 s time windows for the following frequency bands: Delta: 1–3.5 Hz, theta: 4–7.5 Hz, alpha1: 8–10 Hz, alpha2: 10.5–12 Hz, beta1: 12.5–15 Hz, beta2: 15.5–25 Hz, and gamma: 25.5–45 Hz. To obtain a measure of the power spectral density (PSD) FFT values were squared and all FFT bins within a frequency band range were averaged. EEG PSD was calculated for each participant, condition, electrode, and frequency band. The resulting PSD data set consisted of the dimensionality of 2 conditions, 64 electrodes, and 7 frequency bands. In addition, to achieve a reduction level, we averaged the values across all electrodes, resulting in the global band power (1–45 Hz).

### Statistics

To calculate correlations between the EEG features and the HSPS-G summary score as well as subscales, Spearman’s rank correlation was applied after determining that the distribution was not appropriate for parametric testing by the Shapiro–Wilk test. Correlations were calculated from the mean of the time series across participants. After trichotomizing the scale, the lowest and the highest 30% of the sample were contrasted as polar opposites. Thus, the sample was grouped in regard to the HSPS-G summary score into highly sensitive persons (HSP, 78–104) and low sensitive persons (LSP, 0–43) participants (Lionetti et al., 2018). For a comparison between the groups, effect sizes of the temporal mean of each frequency band were calculated for each electrode defined as standardized mean differences (Cohen’s *d*; Cohen, 2013). These were submitted to a paired two-tailed *t*-test. At the global band power level, there are a total of seven frequency bands and two comparisons, leading to 14 variables. In the spatially resolved data with 64 electrodes, statistics yield 896 variables, indicating that 44 values should randomly achieve significance at a 0.05 level. Correction for multiple comparisons is not trivial as the variables s may be dependent on each other. Here we have applied false discovery rate (FDR) adjustment to the resulting *p*-values, which measures the proportion of false discoveries among all discoveries (Benjamini and Hochberg, 1995). FDR adjustment was applied on all three dimensions, i.e., on the level of 7 frequency bands, 64 electrodes, and 2 comparisons. Significance was set at *p* < 0.05.

## Results

The recruited 115 participants ranged from 18 to 64 years (M = 33.1, SD = 13.3), 71.3% of whom were women. 38.3% of the sample consisted of psychology students. Detailed sociodemographic data can be found in Table 1. The first group (highly sensitive persons, HSP) consisted of n = 47 participants (mean age 41.75 ± 12.7 years, 24 females/ 23 males), with a mean HSPS-G summary score of 85.14 ± 7.7. The second group (low sensitive persons, LSP) comprised n = 32 participants (mean age 38.15 ± 5.1 years, 20 females/ 12 males) with a mean HSPS-G summary score of 22.97 ± 10.35. The groups did not differ statistically significant regarding age (*p* = 0.869), sex (*p* = 0.649), the current living situation (*p* = 0.586), education (*p* = 0.593), and job status (*p* = 0.632; Table 1).

**Table 1:** Sociodemographic data (*n* = 115).

	Total *n* (%)	HSP *n* (%)	LSP *n* (%)
	115	47	32
Age (years)	33.1 ± 13.3	41.75 ± 12.7	38.15 ± 5.1
Sex			
Male	32 (27.8)	23 (48.9)	12 (37.5)
Female	82 (71.3)	24 (51.1)	19 (59.4)
Diverse	1 (0.9)		1 (3.1)
Living situation			
Alone	25 (21.7)	10 (25.6)	5 (12.5)
With partner/family	48 (41.7)	22 (56.4)	12(30.3)
Flat-sharing community	8 (7)	4 (10.3)	2 (5.0)
Relationship yes	76 (66.1)	28 (71.8)	26 (65.0)
Education			
Secondary school certificate	13 (11.3)	9 (23.1)	2 (5.0)
Baccalaureate	45 (39.1)	8 (20.5)	21 (52.5)
Bachelor	24(20.9)	4 (10.3)	12(30)
Master	9 (7.8)	4 (10.3)	2 (5.0)
Diploma	10 (8.7)	5 (12.8)	2 (5.0)
PhD	1 (0.9)	1 (2.6)	-
Other	13 (11.3)	8 (20.5)	1 (2.5)
Job			
Self-employed	1 (9.6)	7 (17.9)	1 (2.5)
Employee	39 (33.9)	21 (53.8)	7 (17.5)
Stay at home	1 (9)	1 (2.6)	-
Student	48 (11.3)	4 (10.3)	3 (7.5)

After trichotomizing the Highly Sensitive Person Scale (HSPS-G), the lowest and the highest 30% of the sample were contrasted as polar opposites and divided into the groups high sensitive persons (HSP) and low sensitive persons (LSP).

### Correlations between sensory processing sensitivity and EEG spectral features

To evaluate whether there is a significant association between the self-reported level of SPS with EEG features, a Spearman’s rank correlation was estimated. During the resting state with eyes open the summary score of the HSPS-G correlated positive with global power (*Spearman’s* ρ = 0.22, *p* < 0.05), beta 1 (*Spearman’s* ρ = 0.20, *p* < 0.05), beta 2 (*Spearman’s* ρ = 0.24, *p* < 0.01), and gamma (*Spearman’s* ρ = 0.25, *p* < 0.01). Positive correlations with these frequency bands were also found with the subscales LST, EOE and AES (Table 2). However, during the resting state with eyes closed, the summary scores did not significantly correlate with any of the EEG spectral parameters (Table 3). In addition, neither age, sex, current living situation, education, nor job status was found to be statistically significantly correlated with any of the frequency bands.

**Table 2:** Spearman correlations of spectral parameter from the mean of the time series of the eyes open resting state across participants after averaging over channels; *n* = 115.

rho	‘HSPS-G_SUM’	‘HSPS-G_EOE’	‘HSPS-G_LST’	‘HSPS-G_AES’
Delta	−0.089	−0.105	−0.106	−0.021
Theta	−0.041	−0.086	−0.054	0.063
Alpha1	0.050	0.036	0.043	0.070
Alpha2	0.005	0.008	−0.023	0.087
Beta 1	0.198*	0.161	0.185*	0.195*
Beta 2	0.242**	0.202*	0.210*	0.269**
Gamma	0.249**	0.215*	0.214*	0.243**
Global	0.216*	0.178	0.184	0.235*

‘HSPS-G_SUM’ = summary score of the German highly sensitive person scale, ‘HSPS-G_EOE’ = subscale ease of excitation of the German highly sensitive person scale, ‘HSPS-G_LST’ = subscale aesthetic sensitivity of the German highly sensitive person scale, ‘HSPS-G_AES’ = subscale low sensory threshold of the German highly sensitive person scale.

** *p* < 0.01.

* *p* < 0.05.

**Table 3:** Spearman correlations of spectral parameter from the mean of the time series of the eyes closed resting state across participants after averaging over channels; *n* = 115.

rho	‘HSPS-G_SUM’	‘HSPS-G_EOE’	‘HSPS-G_LST’	‘HSPS-G_AES’
Delta	−0.129	−0.118	−0.146	−0.077
Theta	−0.098	−0.093	−0.119	−0.033
Alpha1	−0.047	0.008	−0.081	−0.021
Alpha2	−0.029	0.054	−0.104	0.035
Beta 1	0.125	0.131	0.084	0.153
Beta 2	0.176	0.183	0.131	0.195*
Gamma	0.110	0.099	0.091	0.089
Global	0.093	0.108	0.048	0.114

‘HSPS-G_SUM’ = summary score of the German highly sensitive person scale, ‘HSPS-G_EOE’ = subscale ease of excitation of the German highly sensitive person scale, ‘HSPS-G_LST’ = subscale aesthetic sensitivity of the German highly sensitive person scale, ‘HSPS-G_AES’ = subscale low sensory threshold of the German highly sensitive person scale.

* *p* < 0.05.

### Group comparisons

**Figure 1:**
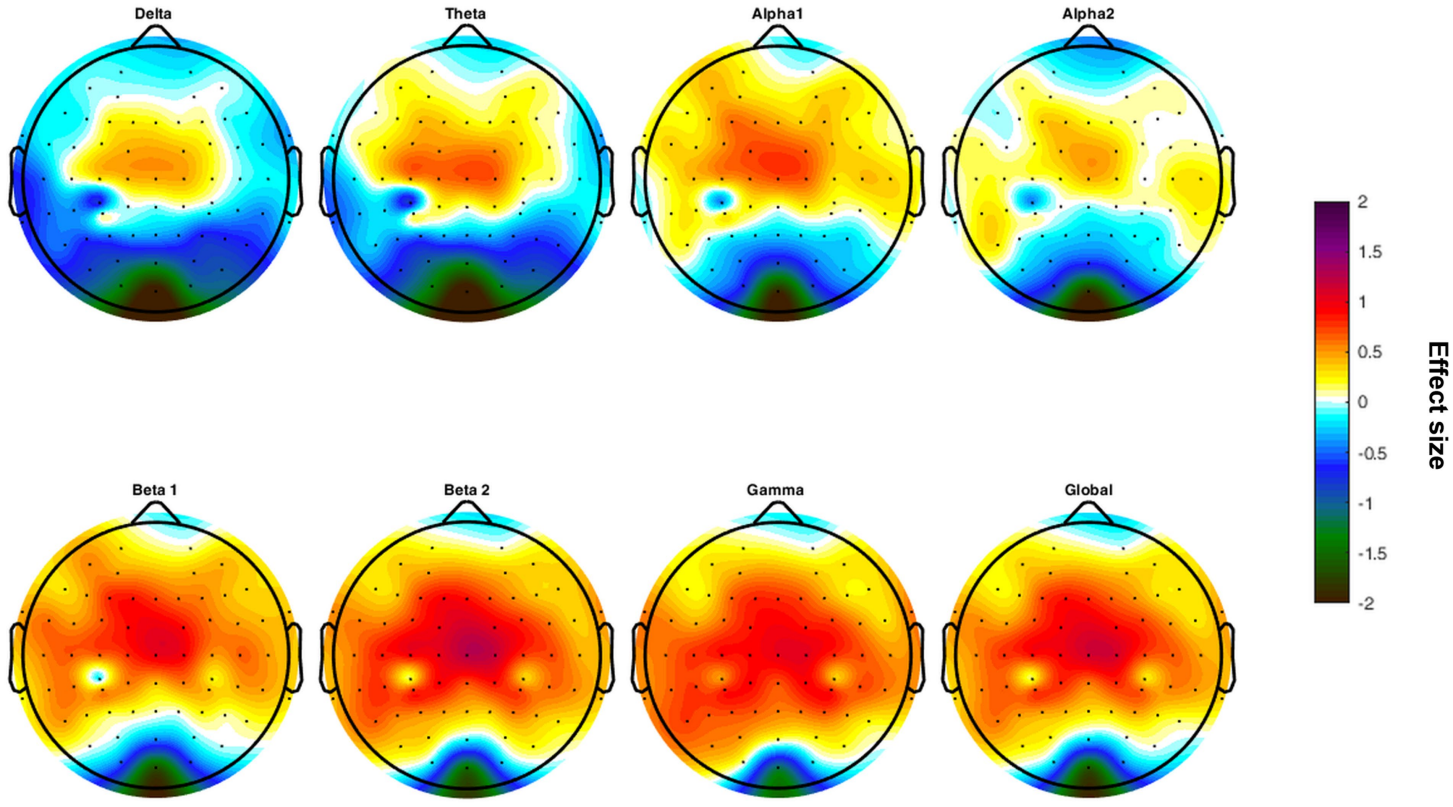
Topographical maps of differences in the effect size calculated for each frequency band comparing the groups of highly sensitive persons and low sensitive persons.

The HSP group revealed higher beta 2 power (4.38 ± 0.32 vs. 4.21 ± 0.17, *p* = 0.014), higher power in the gamma frequency band (4.21 ± 0.37 vs. 4.00 ± 0.25, *p* = 0.010) as well as increased global EEG power (4.38 ± 0.29 vs. 4.25 ± 0.17, *p* = 0.041; Table 4). The higher EEG activity in the HSP group was most pronounced in the central, and parietal, region, whereas lower EEG activity was most present in occipital areas (Figure 1).

**Table 4:** Comparison of the temporal mean of the power spectral density for each frequency band during a task-free resting state between the highly sensitive and the low sensitive group with a paired two-tailed *t*-test.

Power spectral density	Highly sensitive persons (HSP)	Low sensitive persons (LSP)	*p*-value
Delta	5.09 ± 0.20	5.12 ± 0.16	0.061
Theta	4.57 ± 0.19	4.59 ± 0.15	0.072
Alpha 1	4.52 ± 0.23	4.52 ± 0.23	0.091
Alpha 2	4.55 ± 0.27	4.57 ± 0.27	0.090
Beta 1	4.49 ± 0.20	4.45 ± 0.16	0.061
Beta 2	4.39 ± 0.28	4.15 ± 0.26	0.014*
Gamma	4.12 ± 0.35	4.04 ± 0.35	0.010*
Global	4.41 ± 0.26	4.28 ± 0.24	0.041*

* *p* < 0.05.

## Discussion

The present study aimed to investigate neurophysiological correlates associated with the level of Sensory Processing Sensitivity (SPS) in a sample of 115 subjects. The participants were divided into highly sensitive and low sensitive groups based on their HSPS-G summary scores, and their EEG activity was compared. The analysis revealed a significant difference in global activity, as well as in the beta 2 and gamma frequency bands, between the two groups. Interestingly, this effect was observed only during a task-free resting state with eyes open, but not during eyes closed. This finding may indicate a deeper level of cognitive information processing in Highly Sensitive Persons (HSPs). In line with this assumption, previous studies have demonstrated that HSPs perform better in visual search tasks, despite reporting higher levels of subsequent stress compared to non-HSPs. The authors explained this superior performance by attributing it to increased activation of working memory in HSPs (Gerstenberg, 2012). This interpretation aligns with the observed increase in gamma power, as experimental research has shown that gamma band response plays a crucial role in both working and long-term memory (Gruber et al., 2004; Jensen et al., 2007). Furthermore, it has been suggested that the thalamic filter, which determines the relevance of incoming stimuli, categorizes more stimuli as relevant in HSPs compared to non-HSPs (Jagiellowicz et al., 2011). As a result, HSPs perceive and process environmental stimuli in greater detail, leading to a broader range of perceived and received stimuli, but also to chronic stress experiences (Jagiellowicz et al., 2011; Aron et al., 2012).

Additionally, the study identified that the higher EEG activity in the HSP group was most pronounced in the central, parietal, and temporal regions. This observation may indicate increased activation of brain areas involved in high-order visual processing, consistent with findings from an fMRI study conducted by Jagiellowicz et al. (2011). Further fMRI research has also demonstrated that SPS is associated with enhanced connectivity within regions of the ventral attention, dorsal attention, and limbic networks (Acevedo et al., 2021), which supports the results presented in this study.

The main practical implications of these findings include advancements in clinical diagnostics, particularly in differentiating across a spectrum of psychological disorders that are associated with abnormal information processing, such as attention deficit-hyperactivity disorder (ADHD) and autism (Wang et al., 2013; Newson and Thiagarajan, 2018). For example, the theta/beta ratio under the eyes closed resting condition has been proposed and approved by the FDA as a diagnostic biomarker for ADHD in children (Newson and Thiagarajan, 2018). This study represents an initial step towards achieving similar diagnostic markers for SPS, provided that validation and consistency can be confirmed in other samples.

### Limitations

This study has several limitations worth noting. Firstly, the sample obtained *ad-hoc* consisted of 115 participants, which was heterogeneous in nature (71.3% women; 38.1% psychology students). Notably, in the HSP group, the majority of participants were female, while in the LSP group, the proportion of male subjects was 65.7%. It has been suggested that women tend to score higher on the HSPS-G and identify more with the construct of SPS (Greven et al., 2019; Konrad and Herzberg, 2019). Recruitment occurred through the High Sensitivity Research Association groups,^1^ forums for highly sensitive individuals, and email distribution lists of HSP coaches. Hence, it cannot be ruled out that study participants were already aware of the SPS trait and the associated questionnaire, which might have influenced their responses based on knowledge rather than personal experience.

Another limitation arises from the construction of the HSPS-G (Konrad and Herzberg, 2019). The scale comprises solely positively worded items, potentially leading to acquiescence tendencies among participants. This response style could result in more frequent response profiles that overestimate the presence of actual HSP in the sample. A potential solution for future research would be to invert some items. Additionally, the HSPS-G is based on Aron and Aron’s original 1997 scale, which has remained unchanged since then (Aron and Aron, 1997). Over the past 20 years, there has been a significant increase in research interest regarding SPS, necessitating an adapted measurement instrument that also encompasses the positive aspects of SPS (Greven et al., 2019).

Furthermore, it is important to consider the extent to which the HSPS-G truly measures SPS, as there is significant content overlap with psychopathological symptoms from the neurotic, stress, somatoform, and affective disorder spectra. Another critical point is that the group classification (HSP vs. LSP) was based on extreme groups (30%). This categorization was implemented to allow for a more nuanced examination of trait expression differences in SPS. However, the present study cannot definitively determine when a person should be considered HSP or LSP, as this has not been conclusively established in previous research (Greven et al., 2019). Moreover, the group division limits the generalizability of the results and the comparison with existing studies. For this reason, the study not only reported extreme group comparisons but also provided correlative results.

Finally, it is worth noting that the different investigators involved potentially could have influenced the highly sensitive subjects, as HSPs can be strongly influenced by people’s moods or emotions (Jagiellowicz et al., 2011). To minimize corresponding biases, the investigators underwent extensive training and received detailed instructions on how to interact with HSP individuals.

### Practical implications and future research

Further neurophysiological studies are required for validation and testing of consistency. In addition, there are many implications for future research. Exemplarily, individual sensory channels (e.g. auditory, olfactory, tactile, gustatory) should be stimulated in a controlled setting during neurophysiological assessment. This could test whether HSP also respond more strongly to other sensory input, or whether perhaps different HSP types exist with respect to sensory stimulus processing. In addition, it would be interesting to see whether neurophysiological studies show specific patterns of stimulus overload in the brain of HSP compared to LSP above a certain stimulus threshold. The question arises whether HSP switch into an automatic protection or coping mechanism after a certain stimulus threshold. Also, psychopathologies should be measured with clinical interviews and standardized questionnaires in future studies in order to control for them and to better compare different psychopathologies in electrophysiological studies with HSP. Here, future studies should control the influence of factors such as age, gender, medication use, stress levels, smoking, and levels of fatigue. The findings of the current study have practical implications in psychotherapeutic practice. Developing psychoeducational interventions for individuals with high levels of SPS can assist patients in gaining a better understanding and acceptance of their innate distinct neural stimulus processing, rather than pathologizing it. Consequently, patients may become more adept at distinguishing between symptoms of their mental illness and those stemming from stimulus overload. In the realm of psychotherapy, stress reduction techniques that foster resilience, such as meditation or autogenic training, should be implemented. Furthermore, it is crucial to prioritize enhancing the self-efficacy of individuals with mental health conditions and high sensitivity levels by acknowledging sensitivity-related needs. This can involve addressing specific workplace conditions or accommodating unique requirements during medical examinations. The study’s findings suggest that Highly Sensitive Persons (HSPs) exhibit heightened sensitivity to environmental stimuli. Therefore, it is essential to investigate how to optimize the handling and environmental conditions for this patient group, particularly in clinical settings such as doctors’ offices or clinics. By doing so, HSPs may experience reduced stress levels and potentially even less pain during medical examinations.

## Conclusion

The present study provides, for the first time, neurophysiological signatures associated with Sensory Processing Sensitivity (SPS). These findings have potential implications, among others, for developing biomarkers for clinical diagnostics to differentiate between psychopathologies and for monitoring the effectiveness of therapeutic interventions. The study provides evidence that neural processes differ between Highly Sensitive Persons (HSPs) and Low Sensitive Persons (LSPs). Specifically, high levels of SPS were found to be significantly associated with increases in high beta and gamma frequency power, as well as increased global EEG power during a task-free resting state with eyes open, but not during resting with eyes closed. These findings support the central theoretical assumption of enhanced information processing in HSPs.

## Data availability statement

The raw data supporting the conclusions of this article will be made available by the authors, without undue reservation.

## Ethics statement

The studies involving human participants were reviewed and approved by Institutional Review Board of the University of the Bundeswehr Munich. The patients/participants provided their written informed consent to participate in this study.

## Author contributions

NM-S and NW: conceptualization and writing—original draft preparation. NM-S and NW, PK, TH, and JK: methodology. NW and TH: formal analysis. NM-S, NW, PK, TL, TH, and JK: investigation. NM-S and PK: data curation. PK, JK, TH, and TL: writing—review and editing. JK and TL: supervision. All authors contributed to the article and approved the submitted version.

## Conflict of interest

The authors declare that the research was conducted in the absence of any commercial or financial relationships that could be construed as a potential conflict of interest.

## Publisher’s note

All claims expressed in this article are solely those of the authors and do not necessarily represent those of their affiliated organizations, or those of the publisher, the editors and the reviewers. Any product that may be evaluated in this article, or claim that may be made by its manufacturer, is not guaranteed or endorsed by the publisher.

## Footnotes

^1^https://www.hochsensibel.org
